# Structural aspects of displacive transformations: what can optical microscopy contribute? Dehydration of Sm_2_(C_2_O_4_)_3_·10H_2_O as a case study

**DOI:** 10.1107/S2052252517008624

**Published:** 2017-07-04

**Authors:** Alexander A. Matvienko, Daniel V. Maslennikov, Boris A. Zakharov, Anatoly A. Sidelnikov, Stanislav A. Chizhik, Elena V. Boldyreva

**Affiliations:** a Institute of Solid State Chemistry and Mechanochemistry, Siberian Branch of the Russian Academy of Sciences, Kutateladze Street 18, Novosibirsk 630128, Russian Federation; b Novosibirsk State University, Pirogova Street 2, Novosibirsk 630090, Russian Federation

**Keywords:** thermomechanical effects, solid-state chemical reactions, martensitic transformations, topotactic transformations, materials modelling, phase transitions, crystal morphology, properties of solids, optical microscopy

## Abstract

For martensitic transformations the macroscopic crystal strain is directly related to the corresponding structural rearrangement at the microscopic level. *In situ* optical microscopy observations of the interface migration and the change in crystal shape during a displacive single crystal to single crystal transformation can contribute significantly to understanding the mechanism of the process at the atomic scale. This is illustrated for the dehydration of samarium oxalate decahydrate in a study combining optical microscopy and single-crystal X-ray diffraction.

## Abstract 2

A comparison is made of information on the structural aspects of the displacive dehydration of samarium oxalate decahydrate obtained from optical microscopy observations and X-ray diffraction studies.

## 1. Introduction   

An intrinsic difference between a liquid and a solid is that a solid has a shape, which resists any attempt to change it. A chemical transformation necessitates a change in atomic positions (*i.e.* the structure at the microscopic level), and this serves as the origin of mechanical stress. This mechanical stress can subsequently relax through various channels, including fracture, plastic deformation or a change in macroscopic crystal shape (Chupakhin *et al.*, 1987 ▸). Recent years have seen an explosive interest in various ‘mechanical’ effects in individual molecules, as recognised by the 2016 Nobel Prize in Chemistry awarded to J.-P. Sauvage, J. F. Stoddart & B. L. Feringa, as well as in crystalline and non-crystalline solids. The latter include bending, twisting and jumping of samples (Naumov *et al.*, 2015 ▸).

Any macroscopic mechanical effect accounts for microscopic structural changes, but a relation between the two is not always straightforward. There is, however, a certain class of structural transformations – martensitic phase transitions – for which microscopic strain is clearly related to the orientation of the propagating interface and changes in crystal shape (Delaey, 2001 ▸; Christian, 2002 ▸). During a martensitic transformation, atoms move coherently and cooperatively with high velocity, only weakly dependent on temperature. Such a transformation is always accompanied by the presence of orientation relationships between the phases and a macroscopic change in shape of the transformed region (Kelly, 2006 ▸).

Most examples of martensitic transformations refer to structural phase transitions. It has long been considered that martensitic transitions must be diffusionless and therefore cannot include a change in chemical composition (Christian, 2002 ▸). However, a few chemical transformations have been documented more recently, which are accompanied by structural transformations similar to those seen for martensites. Such examples have been termed diffusional-displacive (Zhang & Kelly, 2009 ▸). The bainite transformation of steel (Bhadeshia, 2001 ▸), decomposition of solid solutions (Howe *et al.*, 1985 ▸) and oxidation of tantalum (Wayman & Landuyt, 1968 ▸) serve as excellent examples.

Martensitic transformations were first reported for metals and alloys, where they play a central role in metal processing (Christian, 1975 ▸). Early examples were also found in minerals, underpinning many geochemical processes (Carpenter *et al.*, 1998 ▸), in inorganic ceramic materials (Kriven, 1988 ▸), in organic crystals (Görbitz *et al.*, 2016 ▸; Anwar *et al.*, 2007 ▸; Jones *et al.*, 1975 ▸; Panda *et al.*, 2014 ▸, 2015 ▸, 2016 ▸; Birkedal *et al.*, 2002 ▸; Vatulev & Prikhot’ko, 1965 ▸; Sahoo *et al.*, 2013 ▸; Yangui *et al.*, 2015 ▸; Naumov *et al.*, 2015 ▸), and have even been found to occur in proteins and viruses over the course of biological processes (Olson & Hartman, 1982 ▸). Thus, martensitic displacive transformations belong to one of the most general phenomena in condensed hard and soft matter science.

It is not usually possible to observe the cooperative motions of atoms that underpin macroscopic transformations with the naked eye. However, single crystals of molecular compounds can provide this unique opportunity. For example, a comparison of the brittle and flexible behaviour of molecular crystals by mere observations (Reddy *et al.*, 2010 ▸, 2006 ▸) can help distinguish between different types of intermolecular interaction, with these preliminary conclusions perfectly matching direct structural analysis (Panda *et al.*, 2015 ▸).

A single crystal can distort its shape during a cooperative displacement of chemical species, but remain intact. Such a scenario is favoured by the existence of a network of hydrogen bonds that can act as springs, distorted when strained and restored after the strain is removed (Kolesnik *et al.*, 2005 ▸; Goryainov *et al.*, 2005 ▸; Tumanov *et al.*, 2008 ▸; Boldyreva *et al.*, 2006 ▸; Zakharov & Boldyreva, 2013 ▸, 2014 ▸; Zakharov *et al.*, 2015 ▸; Losev *et al.*, 2016 ▸). By measuring the angles between the crystal faces before and after the transformation, as well as the orientation of the transformation interface with respect to the crystal faces during the transformation, one can derive information related to the unit-cell parameters of the transformation product. In some sense, this is another version of the classical Haüy approach when angles between crystal faces are correlated with internal crystal structure (Kunz, 1918 ▸; Shaskol’skaya & Shafranovskii, 1981 ▸; Haüy, 1784 ▸). Knowing how a unit cell changes during a transformation, the orientation relationships between the crystallographic axes of the reactant and the product, and the starting crystal structure, one can suggest a model of atomic displacements that can account for such changes. This approach is used when analysing topotactic solid-state transformations (Günter & Oswald, 1975 ▸; Figlarz, 1990 ▸). Phase transitions in inorganic materials and minerals on varying the temperature (Angel *et al.*, 2013 ▸; Waeselmann *et al.*, 2012 ▸) and pressure (Mihailova *et al.*, 2015 ▸; Angel & Bismayer, 1999 ▸; Angel *et al.*, 2004 ▸) have been described in terms of cooperative displacements, but it was not possible to watch a single crystal to single crystal transformation with the naked eye, the information about all the orientation relations being derived from X-ray diffraction data.

In the present work we report for the first time an obvious example of a martensitic transformation accompanying a dehydration of a molecular salt. Under certain conditions, the process preserves a single crystal intact. This enabled us to gain substantial information on the structure of the product from the analysis of the changes in the crystal shape, and then to verify and refine the proposed model based on an independent single-crystal X-ray diffraction analysis. We could also get an insight into the mechanism of the transformation by direct *in situ* observation of the propagation of the interface separating the reactant and the product phase.

## 2. Experimental   

### 2.1. Crystal growth   

Crystals of Sm_2_(C_2_O_4_)_3_·10H_2_O were produced by slow mixing of aqueous 1% solutions of samarium nitrate (reagent grade) and oxalic acid (reagent grade) at 60°C. Plate-like crystals elongated along the *c* axis, with the largest faces (010) (predominantly) or (100) (minor fraction), were obtained.

### 2.2. Single-crystal X-ray diffraction   

The crystal structure of Sm_2_(C_2_O_4_)_3_·10H_2_O was solved using single-crystal X-ray diffraction data using an Oxford Diffraction Gemini R Ultra diffractometer with a CCD detector and Mo *K*α radiation. Parameters characterizing the data collection and refinement of the crystal structure are summarized in Table S1 in the supporting information. The structure was solved by direct methods using the software package *SHELXS* (Sheldrick, 2008 ▸) and refined using *SHELXL* (Sheldrick, 2015 ▸), with *X-STEP32* (Stoe & Cie, 2000 ▸) as the graphical user interface. The positions of the hydrogen atoms of the two outer-sphere water molecules disordered over four positions have not been determined. For all other water molecules, the hydrogen atoms were first found from difference Fourier maps and then refined with a restraint of 0.9 Å on the O—H distance, with a standard deviation of 0.05 Å. The values of the isotropic thermal displacement parameters for the hydrogen atoms were set as 150% of the *U*_eq_ of the corresponding oxygen atom to which the hydrogen atom belongs. The orientation of the crystal edges with respect to the crystallographic axes was determined using *CrysAlisPro* (Rigaku Oxford Diffraction, 2016 ▸) software.

The main features of the structural model for the de­hydration product, Sm_2_(C_2_O_4_)_3_·6H_2_O (unit-cell parameters and atomic coordinates), were first proposed based on optical microscopy observations (see *Results and Discussion* section, as well as the supporting information). The structure was then solved independently by single-crystal X-ray diffraction analysis of a single-crystalline fragment of the product phase preserved after careful dehydration. Data collection, crystal structure solution and refinement were performed in the same way as for Sm_2_(C_2_O_4_)_3_·10H_2_O. Parameters characterizing the data collection and refinement of the crystal structure are summarized in Table S1 in the supporting information.

*PLATON* (Spek, 2009 ▸) was used for visualization, analysis and quality control of the crystal structure determinations. The strain ellipsoid parameters were calculated based on the cell parameters of the parent and product phases.

Structural data were deposited in the form of CIF files in the Cambridge Structural Database (Groom *et al.*, 2016 ▸), with identification numbers CCDC 1521443 for Sm_2_(C_2_O_4_)_3_·10H_2_O and 1521444 for Sm_2_(C_2_O_4_)_3_·6H_2_O. These can be downloaded free of charge from http://www.ccdc.cam.ac.uk.

### 2.3. Optical microscopy   

*In situ* observations of crystal dehydration were carried out using a POLAM-213 (LOMO) optical microscope with a custom-built heating stage, including a transparent heating element (sputtered conductive layer of tin dioxide on a thin glass plate). Heating was done either in air or in silicone oil at a rate of 1–5°C min^−1^. The solid-to-solid transformation started at ∼60°C in air or at ∼80°C in silicone oil. A Nikon D7100 camera was used for photo and video recording. The unit-cell parameters of the dehydration product, Sm_2_(C_2_O_4_)_3_·6H_2_O, and the atomic coordinates were first proposed based on optical microscopy observations of the changes in crystal shape (see *Results and Discussion* section, and a more detailed step-by-step description of the algorithm in the supporting information). *In situ* observations of the propagation of the interface between the reactant and product single-crystalline fragments, in particular the orientation of the interface, enabled us to elucidate the mechanism of the transformation and the structure of the interface.

### 2.4. Thermogravimetry (TG) measurements   

The dehydration of powder samples of Sm_2_(C_2_O_4_)_3_·10H_2_O was studied by thermogravimetry (SETARAM B70 and NETZSCH STA 449F1).

## 3. Results and discussion   

### 3.1. Crystal structures of lanthanide(III) oxalates   

The decahydrates Ln_2_(C_2_O_4_)_3_·10H_2_O with Ln = La–Er (including yttrium) crystallize in the monoclinic system, space group *P*2_1_/*c* (Hansson, 1970 ▸; Hansson * et al.*, 1968 ▸; Ollendorff & Weigel, 1969 ▸; Huang *et al.*, 1991 ▸) and consist of layers built up by the packing of honeycomb hexagonal six-membered rings to form a metal–oxalate network. Decahydrate crystal structures Ln_2_(C_2_O_4_)_3_·10H_2_O (Ln = La, Ce, Pr, Nd, Sm, Eu, Gd, Tb and Dy) were first published by Ollendorff & Weigel (1969 ▸); the difficulty in locating most of the outer-sphere water molecules, which represent 40% of the water content in the stoichiometric formula, was noted. In later publications, crystal structure refinement has been performed for several lanthanides and the outer-sphere interlayer water molecules were suggested to be disordered over four (Wang *et al.*, 2013 ▸), five (Hansson *et al.*, 1973 ▸ and references therein) or seven (Huang *et al.*, 1991 ▸) positions. We are not aware of any papers describing the Sm_2_(C_2_O_4_)_3_·10H_2_O crystal structure.

Heavier Ln = Ho–Lu do not form decahydrates and crystallize as hexahydrates Ln_2_(C_2_O_4_)_3_·6H_2_O, space group 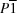 (Hansson, 1973 ▸). The structure of the lanthanide oxalate hexahydrates is closely related to that of the lanthanide oxalate decahydrates. The only major difference is the number of water molecules, which are either in the outer sphere or are linked to the lanthanide. Two water molecules are bound to the lanthanide. Thus, the lanthanide ions are eight-coordinated. The smaller coordination number was thought to be related to lanthanide contraction (Hansson, 1973 ▸). Changes in the coordination polyhedron lead to a slight distortion of the metal–oxalate network. The structure also contains two outer-sphere water molecules per formula unit located between metal–oxalate layers. Another family of layered lanthanide oxalate hexahydrates, free of ‘zeolitic water molecules’, [Ln(H_2_O)_3_]_2_(C_2_O_4_)_3_ with Ln = Eu–Dy, has been described by Trollet *et al.* (1997 ▸). Within this family, the lanthanide cation is nine-coordinated, as in Ln_2_(C_2_O_4_)_3_·10H_2_O. The six-membered ring is still present in the structure but its shape is quite different from that of the decahydrate: instead of being practically hexagonal, it is rectangular. These hexahydrates were obtained by continuous heating (from one week to one month) of a mixture of lanthanide and sodium oxalates at 120–150°C. Sm_2_(C_2_O_4_)_3_·6H_2_O was reported to form as an intermediate product on thermal decomposition of Sm_2_(C_2_O_4_)_3_·10H_2_O, but the crystal structure of this compound was not studied (Fuller & Pinkstone, 1980 ▸; Hussein *et al.*, 2003 ▸). We are not aware of any papers describing the Sm_2_(C_2_O_4_)_3_·6H_2_O crystal structure.

### 3.2. Crystal structure of the parent phase   

**Figure 1:**
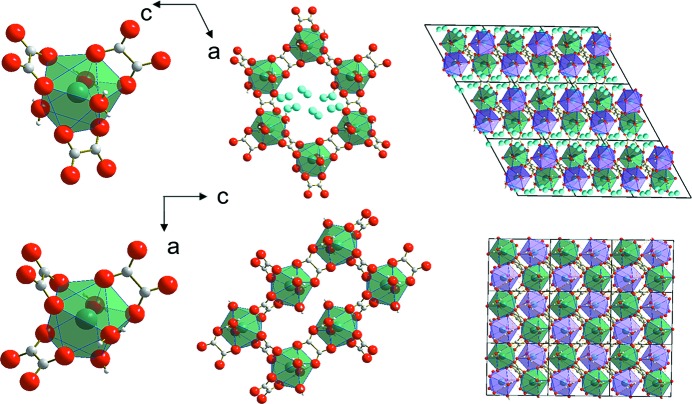
Fragments of the crystal structures of Sm_2_(C_2_O_4_)_3_·10H_2_O (upper row) and Sm_2_(C_2_O_4_)_3_·6H_2_O (lower row).

Sm_2_(C_2_O_4_)_3_·10H_2_O was shown to be isostructural with the decahydrates of the oxalates of other lanthanides (Hansson, 1970 ▸; Ollendorff & Weigel, 1969 ▸; Huang *et al.*, 1991 ▸). The main structural unit in each of these species is the metal–oxalate hexagonal layer (Fig. 1 ▸). The coordination polyhedron around the metal ion includes nine oxygen atoms, six of which belong to oxalate molecules and the other three to H_2_O molecules. Metal–oxalate layers are arranged one above the other with a shift along the crystallographic *c* axis. There are two types of water molecule in the structure: outer-sphere water (two molecules) and inner-sphere water (three molecules) (Table S1 in the supporting information). The samarium ion is coordinated by three inner-sphere molecules of water. Two outer-sphere water molecules are not included in the coordination polyhedron and are located in the interlayer voids between the metal–oxalate layers. These outer-sphere interlayer water molecules are disordered over four positions in the asymmetric unit (Fig. 1 ▸).

### 3.3. Preliminary studies by TG   

TG has shown that heating Sm_2_(C_2_O_4_)_3_·10H_2_O in air leads to the loss of four water molecules at temperatures below 70°C. This results in the formation of a single-phase product, Sm_2_(C_2_O_4_)_3_·6H_2_O. These findings agree with literature reports that the lanthanide oxalate decahydrates usually release four water molecules during the first stage of dehydration, forming hexahydrates (Fuller & Pinkstone, 1980 ▸; Hussein *et al.*, 2003 ▸).

### 3.4. Optical microscopy   

**Figure 2:**
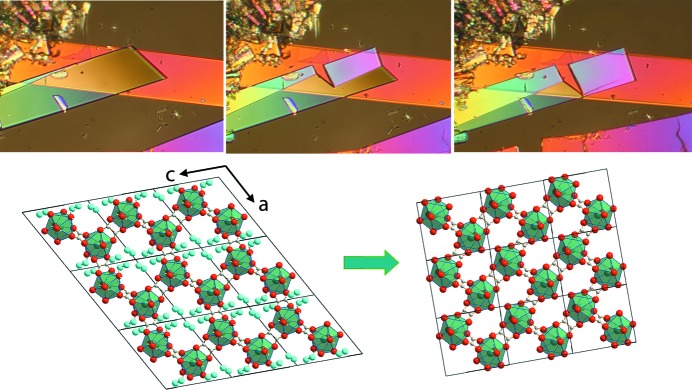
(Top) Optical micrographs of Sm_2_(C_2_O_4_)_3_·10H_2_O during dehydration to Sm_2_(C_2_O_4_)_3_·6H_2_O on heating. (Bottom) The orientation of the fragments of the crystal structures corresponds to the crystal shape. The hexahydrate crystal remains in the same plane. The product structure was calculated from optical microscopy observations and confirmed independently by single-crystal X-ray diffraction (see text).

Observation of the dehydration of Sm_2_(C_2_O_4_)_3_·10H_2_O crystals by optical microscopy revealed a most unusual behaviour: crystals were seen to move, bend, rotate and jump (Video 1 in the supporting information). Obviously, this behaviour is related to the deformation of the crystals over the course of dehydration (Fig. 2 ▸, upper row). In most cases, the crystals cracked to form plate-like particles several micrometres thick. However, some crystals (or parts of crystals) transformed without breaking. Cracking could be suppressed if dehydration was performed under elevated water vapour pressure and if the transformation was slower. To increase the local water vapour pressure over the solid samples, dehydration was performed either in a stream of air at 90% relative humidity or in a drop of silicone oil. The old and new phases transmitted different interference colours if observed in polarized light, and the interface was therefore clearly visible. The interface between the initial and final phase structures formed a small angle with one of the crystal edges and propagated quickly through the crystal. Its movement was accompanied by a change in the crystal shape. The portion of the original crystal that was shaped as a parallelogram became considerably more rectangular (Fig. 2 ▸ upper row, Video 2 in the supporting information). This transformed structure was preserved as a single crystal, as proved by simultaneous extinction of the whole crystal on rotation under plane-polarized light. The extinction position of the initial crystal on rotation around the crystallographic *b* axis co­incided with the *a* axis. The extinction position of the reaction product rotated 15° counterclockwise. The extinction position of the crystals with a developed face (100) coincided with the *b* axis and did not change after the transformation. Since one of the indicatrix axes of the original crystal coincided with the *b* axis, the fact that the extinction position in the reaction product was preserved indicated that the symmetry of the reaction product was preserved along the *b* axis.

The macroscopic change in shape of a transformed region is one of the most striking characteristics of martensitic phase transitions (Christian *et al.*, 1995 ▸). For a single crystal to single crystal transformation, the change in crystal shape is un­ambiguously related to changes in the unit cell. We decided to use this to derive a structural model for the dehydrated product phase from optical observations and the parent crystal structure.

### 3.5. Structural model for the dehydration product derived from optical observations   

The crystal edges of the parent crystal are parallel to the *a* and *c* axes of the monoclinic unit cell (Fig. 2 ▸). The angle between the edges is equal to the monoclinic angle β. Having measured the changes in length of the crystal edges and the angle between them after the transformation, we could estimate the *a*, *c* and β unit-cell parameters of the product phase. In order to estimate the changes in the unit-cell parameter *b*, we followed the dehydration of Sm_2_(C_2_O_4_)_3_·10H_2_O crystals with another crystal habit, with the largest face being the (100) plane. These experiments showed that *b* remains effectively unchanged on transformation. The cell parameters of the dehydration product estimated from optical microscopy are summarized in the supporting information. The principal components of the transformation strain ellipsoid calculated from the cell parameters of the parent and product phases are given in Table S2 (supporting information).

It was assumed that the four water molecules released on dehydration of Sm_2_(C_2_O_4_)_3_·10H_2_O to give Sm_2_(C_2_O_4_)_3_·6H_2_O (as shown by the TG data) are the two outer-sphere water molecules lost per asymmetric unit. As the outer-sphere molecules are removed, the metal–oxalate framework loses its stability; its distortion results in a change in the crystal shape. The strain can be achieved by a shift of metal–oxalate chains stretched along **c** in the [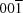] direction by ∼1/2 of the **c** cell vector. This shift does not change the positions of oxalate anions within the chain, but requires the rotation and tilt of oxalate anions connecting the chains. As a result, the faces of coordination polyhedra lying in the shear plane should rotate.

The dehydration studied in this work is an example of a displacive transformation. In general, this type of trans­formation can be described as a combination of ‘homogeneous lattice-distortive strain’ and ‘shuffles’ (Christian *et al.*, 1995 ▸). Changes in the cell parameters and crystal shape are related exclusively to the homogeneous lattice-distortive strain and can be derived from optical observations. Homogeneous strains alone, however, do not always describe the structural transformation completely and really only give the relationship between the positions of the atomic sites defined by the parent and product unit cells. The remaining atoms can be regarded as lying on interpenetrating equivalent lattices or interior points of the corresponding cells and may undergo additional displacements – shuffles – to complete the structural transition from parent phase to product. These shuffles are relative translations of the various subsets of atoms through less than an interatomic distance and have no detectable effect on the shape change associated with the transformation (Zhang & Kelly, 2009 ▸). Shuffles of individual atoms cannot be predicted *a priori* and must be found from X-ray diffraction.

If it is possible to select structural elements of the original structure (layers and chains) that remain almost unchanged during the solid-state transformation and are preserved in the product structure, then deformation of the parent crystal structure can be described as a displacement of these ‘rigid’ structural elements relative to each other. Selecting a rigid element, we fix the mutual arrangement of a group of atoms or, at least, minimize their mutual displacements. This option facilitates the problem, but does not completely exclude the necessity of additional shuffling of atoms to optimize the product structure. Any shuffling of atoms must be compatible with the symmetry of the crystal structure.

In the particular case considered in this work, zigzag chains along the [001] direction formed by coordination polyhedra of samarium can be assumed to be the rigid elements that are preserved through the dehydration (Figs. 1 ▸ and S1). As has been noted previously, crystal structure deformation on transformation can be described as a shift of metal–oxalate chains stretched along **c** in the [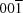] direction by ∼1/2 of the **c** cell vector. One can assume that the mutual arrangement of atoms inside the ‘rigid’ chains does not change significantly during the transformation. On the other hand, it is obvious that the orientation of the oxalate groups that connect the rigid chains can and should change during the transformation. Based on that, the atomic coordinates for two of the three oxalate groups, the samarium atom and two water molecules (containing O8 and O9) were found. The atomic coordinates of the third oxalate group and of the water molecule containing atom O7 were found based on the assumptions that the polyhedron face formed by atoms O1, O2 and O7 rotates as the chains shift with respect to each other, the oxalate group does not change its geometry, and the centre of the C—C bond of this oxalate group is located on the inversion centre.

The procedure is described in detail in the supporting information. Projections of the structure onto (010) are shown in Figs. 2 ▸ and S3, and atomic coordinates are given in Table S3. As one can see, the proposed crystal structure model is fully compatible with the observed crystal shape change (Fig. 2 ▸).

The geometries of the oxalate groups are almost the same in the crystal structures of the decahydrate and the hexahydrate. The distances between the samarium atom and the oxygen atoms in the polyhedron (2.3557–2.6025 Å) in the hexahydrate structure differ significantly from those in the parent structure of the decahydrate (2.4334–2.5508 Å). This indicates that additional optimization of the structure is required, and this can be achieved by slight rotations of the oxalate groups relative to the axes that connect the centre of the oxalate anion and the samarium atom. These shuffles allow one to optimize the crystal structure. Two types of shuffle can be distinguished for this structural transformation. The first type is related to the optimization of the structure and consists of a slight change in the positions of the oxalate groups of the chain, as well as of atoms O8 and O9 of the water molecules. The second type is a radical change in the position of the oxalate anion connecting the chains and the O7 atom of one water molecule.

### 3.6. Structural model for the dehydration product derived from single-crystal X-ray diffraction   

To verify the structural model for the dehydration product, and to find any additional shuffles, as well as the positions of the water molecules in the structure of Sm_2_(C_2_O_4_)_3_·6H_2_O, single-crystal X-ray diffraction data were collected from a fragment of the product crystal. The model derived from the analysis of optical microscopy data was confirmed, and additional data on the space-group symmetry and atomic coordinates were obtained.

**Figure 3:**
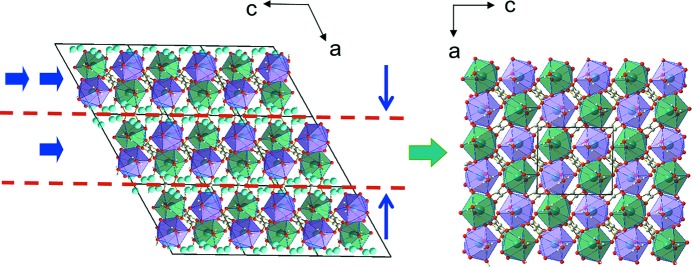
Schematic representation of the Sm_2_(C_2_O_4_)_3_·10H_2_O → Sm_2_(C_2_O_4_)_3_·6H_2_O transformation.

On dehydration of Sm_2_(C_2_O_4_)_3_·10H_2_O to Sm_2_(C_2_O_4_)_3_·6H_2_O the monoclinic space-group symmetry *P*2_1_ */c* is preserved. The cell shape of the metal–oxalate grid is transformed from hexagonal to rectangular (Fig. 1 ▸). The following orientation relationships between the parent and product phases exist: (010)_10_//(010)_6_, [001]_10_//[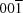]_6_, [010]_10_//[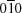]_6_. As was supposed, the outer-sphere water molecules are lost on dehydration and the metal–oxalate grid is distorted. The coordination polyhedron of the metal ion containing nine oxygen atoms is preserved. The positions of the two oxalate anions and two water molecules in the coordination polyhedron do not change on dehydration, whereas one oxalate anion and one water molecule change their positions and orientation (Fig. 1 ▸). In complete agreement with the prediction based on optical microscopy observations, the product structure can be obtained from the structure of the deca­hydrate by shifting layers parallel to the (100) plane along the [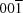] direction by about 1/2 of the *c* parameter, and subsequently contracting the structure in the direction normal to these planes (Fig. 3 ▸). In order to accomplish this shift, one must rotate and tilt the oxalate ions connecting the metal–oxalate chains, which are parallel to the *c* axis. This movement of the oxalate ions causes the displacement of a neighbouring water molecule to a position previously occupied by one of the oxygen atoms belonging to the oxalate ions. As a result, the coordination polyhedron face lying in the plane of the shear is rotated. After the transformation, the shape of a grid cell becomes almost rectangular.

Comparing Figs. 1 ▸, 2 ▸ and S3 (supporting information), one can see that the crystal structure proposed based on the optical microscopy observations of the changes in crystal shape is basically the same as has been determined from single-crystal X-ray diffraction. As might be expected, the major difference is related to the position of the oxalate group connecting chains to each other. In the crystal structure model obtained by single-crystal X-ray diffraction, this oxalate group is rotated by a higher angle from the (010) plane than was predicted based on optical microscopy. The difference in the lattice parameters is related to the limitations of the precision of crystal-shape measurement by optical microscopy.

One can compare the information on the crystal structure that one can get from optical microscopy and that obtained from single-crystal X-ray diffraction measurements.

From optical microscopy we can obtain information about the crystal shape, its optical characteristics (refractive index, extinction in crossed Nicol prisms *etc.*), the morphology of the reaction product, the change in crystal shape during the transformation (shape deformation) and the orientation of the interface. Moreover, optical observations allow us to study the kinetics of the process and follow crystal twinning and fragmentation of the crystal.

Single-crystal X-ray diffraction is a powerful technique for crystal structure determination (lattice parameters, space-group symmetry, atomic coordinates) of a crystal. This technique allows us to determine the orientation relationships of the phases before and after a chemical reaction. Still, this information does not allow us to determine the transformation mechanism (atomic movements, processes related to the propagation of the interface). The presence of the orientation relationships alone is not a proof of the displacive mechanism of transformation (Christian, 2002 ▸). Orientation relationships can also exist for reconstructive transformations (Figlarz, 1990 ▸; Christian, 2002 ▸). Several options of crystal structure changes are also possible for certain orientation relationships (Delaey, 2001 ▸; Zhang & Kelly, 2009 ▸).

Crystal shape deformation is a characteristic feature of martensitic transformations and provides information on the structural strain during a transformation. The shape de­form­ation determines the change in unit-cell parameters during a transformation. The atomic coordinates in the product structure can be found from an analysis of the possible response of the structure corresponding to the observed macroscopic strain. The task is facilitated if it is possible to define structural elements (layers, chains) in the initial structure that are stable during the transformation. Deformation of the structure in this case can be described as displacement of these rigid structural elements relative to each other. This approach may require shuffles to optimize the structure and is useful for creating a primary model of a structure for refinement from a single-crystal or powder X-ray diffraction experiment. A comparison of the possibilities of optical microscopy and single-crystal X-ray diffraction for single-crystal transformation studies is shown in Table 1 ▸.

### 3.7. The orientation of the interface and the structural transformation mechanism   

Optical microscopy observations have proved to be very powerful for suggesting a structural model for the product of single crystal to single crystal dehydration. However, they can do more than that. Optical microscopy observations, in particular the analysis of the precise orientation of the reaction interface with respect to the crystallographic axes, can give an insight into the structure of the interface and the mechanism of transformation. During a martensitic trans­form­ation, atoms at the interface move coherently and co­operatively. This transformation is characterized by a significant shear related to mechanical stresses. These stresses influence the shape of the product particles, the interface orientation and the kinetics of the process. They are also the reason for ‘mechanical response’ effects, such as the thermosalient effect observed also for Sm_2_(C_2_O_4_)_3_·10H_2_O on de­hydration.

According to the phenomenological theory of martensite crystallography (Bowles & Mackenzie, 1954 ▸; Wechsler *et al.*, 1953 ▸), one plane must remain invariant (*i.e.* not change its orientation and not be deformed) as a result of the trans­form­ation. This minimizes the strain energy associated with the transformation. The invariant plane can exist if the signs of the deformation along the two main axes are opposite and the extent of the deformation along the third axis of the strain ellipsoid is small (Christian, 2002 ▸). Only in this case is the relationship between the distortion of the crystal shape and the lattice strain unambiguous. Any deviation from this condition results in generating elastic stresses at the interface separating the parent and product phases, plastic deformation or crystal distortion. In the case of the martensitic dehydration of Sm_2_(C_2_O_4_)_3_·10H_2_O to Sm_2_(C_2_O_4_)_3_·6H_2_O, the principal strain components are −0.344, 0.189 and −0.02 (Table S2 in the supporting information). Structural strain thus satisfies the condition of the presence of the invariant plane when the crystal changes its shape on dehydration.

**Figure 4:**
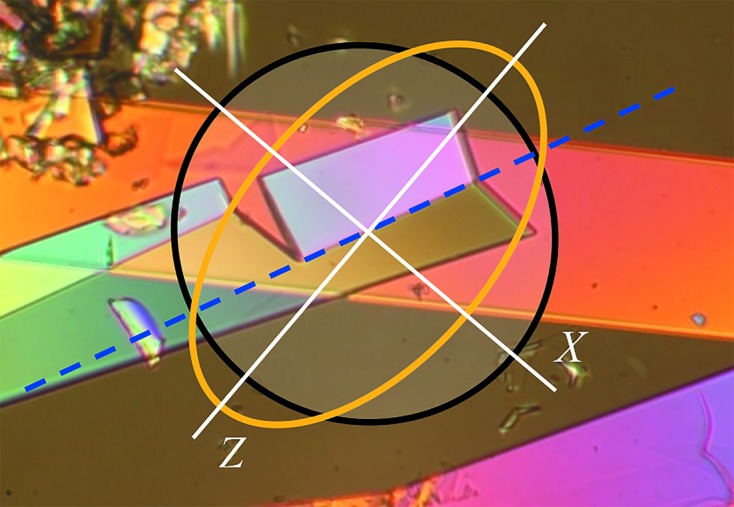
Invariant plane coinciding with the interface (dashed line) and the orientation of the strain ellipsoid during the Sm_2_(C_2_O_4_)_3_·10H_2_O → Sm_2_(C_2_O_4_)_3_·6H_2_O transformation.

The procedure for finding the position of the invariant plane has been described in detail by Bhadeshia (2006 ▸). We have used this procedure in our study (see the supporting information). Fig. 4 ▸ shows a cross section of the strain ellipsoid by plane (010) and the invariant plane (dashed blue line) that forms an angle of about 7° with the *c* axis. The position of the interface observed experimentally matches well with the position of the invariant plane derived from the analysis of structural strain. Such an orientation of the interface corresponds to the minimum strain in the border plane and the minimum strain energy of the structural transformation (Christian, 2002 ▸). This proves that the structural transformation accompanying dehydration is indeed a transformation with an invariant plane and qualifies as a martensitic transformation.

**Figure 5:**
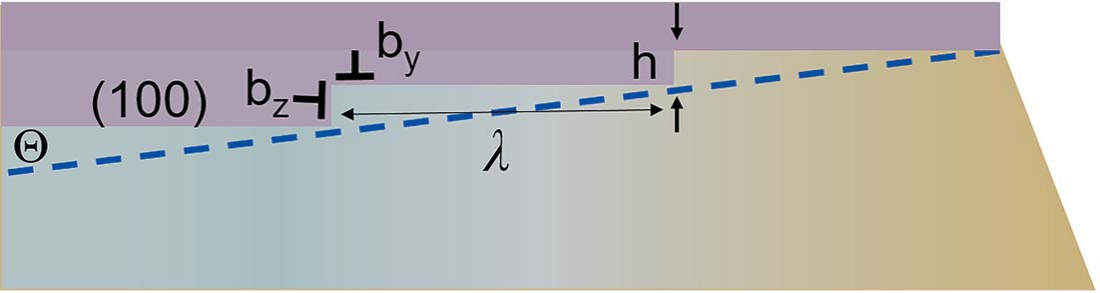
Schematic representation of the structure of the interface, showing (100) terrace segments and disconnections. The macroscopic interface and invariant plane are shown by the dashed line.

Information on the structure of the interface and the mechanism of transformation can be derived based on topological theories (Howe *et al.*, 2009 ▸). Transmission electron microscopy investigations of the structures of martensitic interfaces have revealed the existence of coherent terraces reticulated by arrays of localized interfacial line defects (Ogawa, 2004 ▸; Moritani *et al.*, 2002 ▸). Two types of defect have been found at these interfaces, namely those causing a lattice-invariant deformation, such as slip or twinning dislocations, and transformation dislocations or disconnections (Christian, 2002 ▸). Disconnections combine features of dislocations and steps. They can be characterized by parameters **b** and *h*, where **b** is the Burgers vector and *h* is the step height. The dis­connection motion along an interface accounts for the transfer of material from one phase to the other. In addition, its dislocation character produces a deformation. Thus, dis­connection motion couples deformation with interface migration and is the elementary mechanism underlying displacive transformations (Pond *et al.*, 2003 ▸). Based on optical microscopy observations, we suppose that the interface between the decahydrate and the hexahydrate of samarium oxalate consists of (100) coherent terraces (Fig. 5 ▸). Coherently strained terraces are reticulated by arrays of disconnections with spacing λ. Disconnection motion along an interface causes a shift in the terrace plane along [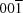], contraction perpendicular to the terrace and the transfer of material from one phase to the other.

The overall interface plane deviation from the terrace plane is defined as tanΘ = 〈*h*〉/〈λ〉, where 〈λ〉 and 〈*h*〉 are the average terrace spacing and the height of the disconnections, respectively. The angle Θ between the interface plane and the (100) face is about 7° (Figs. 4 ▸ and 5 ▸). The average height of a disconnection is equal to the average (100) spacing of deca- and hexahydrate structures, [*d*_100_(10) + *d*_100_(6)]/2 = 9.2884 Å. Thus, the average terrace spacing can be estimated as 75 Å.

One of the postulates of the topological model (Howe *et al.*, 2009 ▸) is the absence of a long-range coherence strain at the interface. This postulate can be described mathematically (Zhang & Kelly, 2009 ▸) as
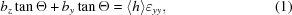
where 〈*h*〉 is the average height of a disconnection (already defined above), Θ is the angle between the terrace plane and interface plane, and ∊_*yy*_ = −0.063 is the strain in the terrace plane due to the difference in atomic spacing between the two phases along [001]. The variables *b_z_* and *b_y_* are the components of the Burgers vector of a disconnection. *b_z_* = *d*_100_(10) − *d*_100_(6) = 1.706 Å corresponds to the difference in step heights between the two phases. The value *b_y_* = 〈*h*〉tan(β_10_ − β_6_) = 5.07 Å is the displacement necessary to transform decahydrate metal–oxalate layers into hexahydrate ones, where β_10_ and β_6_ are the values of the monoclinic angle β for the decahydrate and hexahydrate, respectively. One of the two solutions to this equation, Θ = 6.9°, matches well with the experimentally observed angle of 7° between the interface and the (100) plane (Fig. 4 ▸). Thus, both phenomenological and topological theories of martensitic crystallography predict the interface position correctly. Such an orientation of the interface that corresponds to the invariant plane provides minimum strain at the interface. Overall, crystal deformation can be described by shear (*s*, along the interface) and dilation (δ, volume change, deformation perpendicular to the interface) components. The formation of martensite in a constrained environment must (because of the shape deformation) cause a distortion of the parent lattice in its vicinity. The strain energy due to this distortion, per unit volume of martensite, is approximated by (Bhadeshia, 2006 ▸)
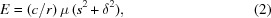
where μ is the shear modulus of the parent lattice and *c*/*r* is the thickness to length ratio of the martensite plate. The product of a martensitic transformation must always have a thin-plate morphology in order to minimize *E*.

The dehydration of Sm_2_(C_2_O_4_)_3_·10H_2_O to form Sm_2_(C_2_O_4_)_3_·6H_2_O is characterized by large values of the shear [*s* = tan(β_10_ − β_6_) = 0.55] and dilation (δ = Δ*V*/*V* = −0.2) components of equation (2). The plasticity of the material is low. Therefore, it is difficult to generate dislocations, their glide is also difficult, and fragmentation is the main channel for the relaxation of mechanical stresses. If the nuclei of the product phase (thin plates growing along [001]) are formed in the crystal bulk, the crystal is destroyed because of large mechanical stress and multiple micrometre-sized platelets are formed. Alternatively, the product phase can nucleate at the surface of the crystal and the interface propagates through the whole crystal; the process is accompanied by a significant change in crystal shape (Fig. 2 ▸, Videos 2 and 3 in the supporting information). In this case, some crystals (or parts of crystals) are transformed without cracking. The reason for this is that nothing hinders a change in the crystal shape if the product nucleates at the surface, and the shape change does not generate elastic strain in adjacent layers. This is the case when we can watch, by optical microscopy, the change in crystal shape resulting from dehydration and draw conclusions on the major features of the crystal structure of the reaction product.

A structural transformation can proceed only if water molecules are removed from the crystal cell. Therefore, the movement of disconnections is accompanied by the diffusion of water molecules to the crystal surface. We assume that the dehydration itself is a diffusional–displacive transformation, and the removal of water and structural rearrangement occur simultaneously at the interface separating the dehydration product from the parent phase. Diffusional–displacive transformations show the distinctive characteristics of both types of transformation – the long-range diffusion required for a reconstructive diffusional transformation and the shape change that is the hallmark of a displacive martensitic transformation (Cohen *et al.*, 1979 ▸). An alternative mechanism of structural transformation could be imagined that includes a two-step dehydration process: (i) removing water molecules from the bulk crystal to form the hexahydrate composition of the crystal and (ii) a subsequent phase transition giving the final hexahydrate crystal structure. In this case, the rate of interface propagation should not depend on the water vapour pressure. This assumption does not match the experimental observations: in the optical microscopy experiment we observe a significant dependence of the process rate on water vapour pressure. The process rate measured based on optical microscopy is in a very good agreement with the TG measurements and the data from phase analysis. These facts prove that water removal and structural transformation are simultaneous processes taking place at the interface. Thus, the structural transformation occurring during dehydration is not a traditional diffusionless martensitic transformation, but belongs in fact to the class of diffusional–displacive trans­form­ations. The dehydration process consists of several stages: removing water molecules from the outer sphere, water diffusion to the crystal surface, water desorption and water diffusion in the gas phase. We can only speculate on the limiting stage of the process and consider water diffusion to the crystal surface as the most probable limiting stage. To find unambiguously which of the stages is rate limiting, one would need to perform a detailed study of the reaction kinetics, *i.e.* to measure the dependence of the interface propagation rate on crystal thickness, temperature, water vapour pressure and gas flow rate.

The structural transformation on dehydration can be compared with those accompanying large-pore/narrow-pore transitions in MIL-type frameworks on including or excluding guest molecules (Giovine *et al.*, 2017 ▸; Reinsch *et al.*, 2016 ▸; Rodriguez *et al.*, 2016 ▸; Salazar *et al.*, 2015 ▸; Schneemann *et al.*, 2014 ▸). The latter are related to elastic ‘breathing’ of the host system depending on the guest size. As for the possibility of preserving single crystals of Sm_2_(C_2_O_4_)_3_·10H_2_O in multiple dehydration–rehydration cycles, this is not straightforward. As can be clearly seen from Video 1 in the supporting information, the ‘free’ samples not covered by oil are fragmented violently on dehydration. Therefore, for such samples it was not possible to observe any propagation of the interface or reversible change in the crystal shape. Dehydration preserving single crystals, or at least their fragments, intact could only be observed for crystals heated under a layer of oil (to increase the partial vapour pressure immediately at the crystal surface and slow down the dehydration). Rehydration under such conditions is not easy.

## 4. Conclusions   

Single-crystal X-ray diffraction is a ‘gold standard’ for any structural study. At the same time, nowadays many solid-state transformations are studied without ever being observed in a microscope, and therefore much information, including information that cannot be obtained otherwise, is lost. Much can be learnt about the structural mechanism of a trans­form­ation by careful watching. We can even, in certain cases, suggest a rather precise structural model based on measurement of the crystal metrics before and after a transformation, if the structure of the parent crystal is known.

The latter holds for displacive solid-state transformations, in particular for martensitic transformations (Cohen *et al.*, 1979 ▸). Such transformations are always accompanied by a change in the shape of the parent crystal, and this distortion always has the characteristics of an invariant-plane strain, when examined at a microscopic scale. The occurrence of such a shape change implies the existence of an atomic correspondence between the parent and product lattices. In such a case, the major features of the crystal structure of the product can in fact be predicted from analysis of the change in crystal shape over the course of the transformation.

Several conditions must be satisfied in order to enable optical microscopy observations to suggest a structural model for the product of a solid-state transformation. The crystal must have a thin-plate morphology, so as to minimize the elastic energy. The nucleation of a new phase should occur at the surface of the parent phase, and not in the bulk, in order to minimize the risk of crystal fragmentation. Plastic deformation should not contribute significantly to the change in crystal shape. This becomes possible when the value of the third principal component of the strain tensor does not exceed 1–2% [as is the case for Sm_2_(C_2_O_4_)_3_·10H_2_O dehydration or for martensitic transformations in most metals]. This value can however be exceeded in the case of less plastic materials due to elastic strain.

Our work illustrates that the crystallographic approach to martensitic transformations can be successfully applied to describe and rationalize diffusional-displacive transformations during solid-state chemical reactions. This approach can be used for reactions where the removal or incorporation of guest molecules leads to a displacive transformation of the crystalline framework. These can be dehydration, desolvation and intercalation of organic and inorganic compounds, formation of metal hydrides, carbides and nitrides, and selected decomposition reactions.

## Supplementary Material

Crystal structure: contains datablock(s) I, II. DOI: 10.1107/S2052252517008624/lt5002sup1.cif

Structure factors: contains datablock(s) I. DOI: 10.1107/S2052252517008624/lt5002Isup2.hkl

Structure factors: contains datablock(s) II. DOI: 10.1107/S2052252517008624/lt5002IIsup3.hkl

Additional tables and figures. DOI: 10.1107/S2052252517008624/lt5002sup4.pdf

Video 1. DOI: 10.1107/S2052252517008624/lt5002sup5.wmv

Video 2. DOI: 10.1107/S2052252517008624/lt5002sup6.mp4

Video 3. DOI: 10.1107/S2052252517008624/lt5002sup7.mp4

CCDC references: 1521443, 1521444

## Figures and Tables

**Table 1:** Comparison of the possibilities of optical microscopy and single-crystal X-ray diffraction for studies of single-crystal transformations

	Optical microscopy	Single-crystal X-ray diffraction
Cell parameters (product)	+	+
Space group symmetry (product)	± (not unambiguously, but some reasonable assumptions are possible; see supporting information)	+
Atomic coordinates (product)	± (for robust structure forming units; see supporting information for further details)	+
Transformation mechanism	+	−
Lattice strain	+	+
*In situ* process observation	+	−
