# Supplementary material for: Structural aspects of displacive transformations: what can optical microscopy contribute? Dehydration of Sm2(C2O4)3·10H2O as a case study
Source: IUCrJ. 2017 Jul 4;4(Pt 5):588–97. doi: 10.1107/S2052252517008624 (PMC5600022; doi:10.1107/S2052252517008624)
Supplement: Supplementary file 4 [file m-04-00588-sup4.pdf]

IUCrJ

Volume 4 (2017)

Supporting information for article:

Structural aspects of displacive transformations: what can optical microscopy contribute? Dehydration of Sm2(C2O4)3·10H2O as a case study

Alexander A. Matvienko, Daniel V. Maslennikov, Boris A. Zakharov, Anatoly A. Sidelnikov, Stanislav A. Chizhik and Elena V. Boldyreva

S1. Experimental structural data

Table S1 Parameters characterizing data collection and refinement of the crystal structures of $Sm_2(C_2O_4)_3{\cdot}10H_2O$ and $Sm_2(C_2O_4)_3{\cdot}6H_2O$. All structures are monoclinic, $P2_1/c$, Z = 4. Experiments were carried out at 293 K using an Oxford Diffraction Gemini R Ultra diffractometer. Absorption was corrected for by the multi-scan methods implemented in *CrysAlis PRO* software. For H-atoms only coordinates were refined.

	$Sm_2(C_2O_4)_3{\cdot}10H_2O$	$Sm_2(C_2O_4)_3{\cdot}6H_2O$
Crystal data		
Asymmetric unit	$Sm(C_2O_4)_{1.5}(H_2O)_3(H_2O)_2$	$Sm(C_2O_4)_{1.5}(H_2O)_3$
M_r	568	496
a, b, c (Å)	11.5863 (5), 9.6080 (2), 10.1371 (2)	8.4368 (3), 9.7963 (3), 9.4986 (4)
β (°)	118.906 (2)	90.393 (3)
V (Å^3)	987.88 (5)	785.04 (5)
Radiation type	Mo $K\alpha$	Mo $K\alpha$
No. of reflections for cell measurement	4549	1917
θ range (°) for cell measurement	2.0–28.2	2.1–28.2
μ (mm^{-1})	5.99	7.51
Crystal size (mm)	0.30 × 0.10 × 0.02	0.12 × 0.10 × 0.01
Data collection		
T_{min}, T_{max}	0.434, 1.000	0.727, 1.000
No. of measured, independent and observed [$I > 2\sigma(I)$] reflections	6384, 2022, 1853	4769, 1595, 1181
R_{int}	0.033	0.035
$(\sin \theta/\lambda)_{max}$ (Å^{-1})	0.625	0.625
Range of h, k, l	h = -14→12, k = -12→11, l = -11→12	h = -10→9, k = -12→10, l = -11→11
Refinement		
$R[F^2 > 2\sigma(F^2)]$, $wR(F^2)$, S	0.022, 0.056, 1.04	0.030, 0.066, 1.04

No. of reflections	2022	1595
No. of parameters	176	136
No. of restraints	31	6
$\Delta\rho_{max}$, $\Delta\rho_{min}$ (e Å^{-3})	0.76, -0.72	1.46, -0.74

Table S2 Principal strain components and orientation of principal axes of the strain ellipsoid with respect to crystallographic axes of the initial structure. The strain ellipsoid corresponds to the $Sm_2(C_2O_4)_3\cdot10H_2O \rightarrow Sm_2(C_2O_4)_3\cdot6H_2O$ displacive transformation.*

Axis	Principal strain	a, °	b, °	c, °
	Calculated from crystal shape change ($\Delta V/V_0$ = -0.22)			
X	-0.34	19.8	90	138.3
Y	0	90	0	90
Z	0.18	70.2	90	48.3
	Calculated from cell parameters ($\Delta V/V_0$ = -0.205)			
X	-0.344	18.6	90	137.6
Y	-0.02	90	0	90
Z	0.189	71.4	90	47.6

* - Cell parameters estimated from the change in the crystal shape observed in an optical microscope: a = 8.55 Å, b = 9.61 Å, c = 9.39 Å, β = 89.5°. The directions of axes ***b*** and ***c*** coincide in the structures of decahydrate and hexahydrate.-Transformation matrix for cell parameters obtained from optical microscopy and single-crystal X-ray diffraction is $\begin{pmatrix} 1 & 0 & 0 \\ 0 & -1 & 0 \\ 0 & 0 & -1 \end{pmatrix}$.

S2. “Structure solution from optical microscopy data”. The algorithm for finding atomic coordinates in the crystal structure of the reaction product, based on the change in crystal shape across the transformation

General considerations

The deformation of the crystal structure, which results from the solid-state transformation, is related to changes in the cell parameters that accompany this transformation. The cell parameters of the product phase were found from optical microscopy observations in a rather straightforward way (see main text). If there were only one atom per Bravais lattice point in the structure, the changes in the unit cell parameters would define the product structure unambiguously. However, when there exist two or more atoms per Bravais lattice point (as is the case in this study), such a simplified approach to modelling the product structure does not work. A uniform deformation of the original atomic structure in accordance with the deformation of the shape of the unit cell would give an incorrect structural model; additional “shuffling” of atoms inside the new unit cell is required. The task of finding the structural model for the product phase can be simplified, if it is possible to select structural elements of the original structure (layers and chains) that remain almost unchanged during the solid-state transformation and are preserved in the product structure. In this case the deformation of the parent crystal structure can be described as a displacement of these "rigid" structural elements relative to each other. Selecting a "rigid" element, we fix the mutual arrangement of a group of atoms or, at least, minimize their mutual displacements. This option facilitates the problem, but does not completely exclude the necessity of additional "shuffling" of atoms to optimize the product structure. Any “shuffling” of atoms must be compatible with the symmetry of the crystal structure.

In the particular case considered in this work, zigzag chains along the [001] direction formed by coordination polyhedral of samarium can be assumed to be the “rigid elements” that are preserved through the dehydration (Figure S1):

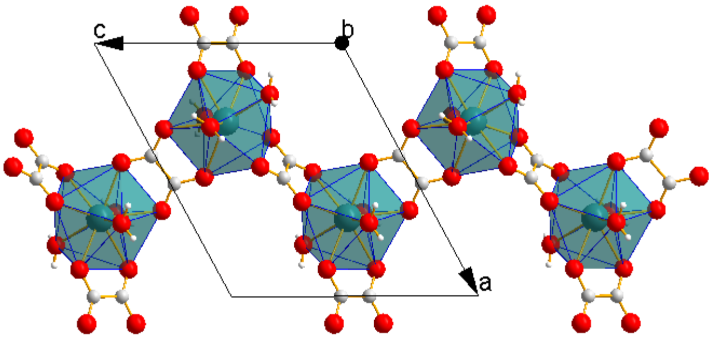

Figure S1 A rigid chain preserved through the dehydration.

These chains are bound to each other by oxalate anions to form a hexagonal network (a “grid”) in the (010) layer (Figure S2):

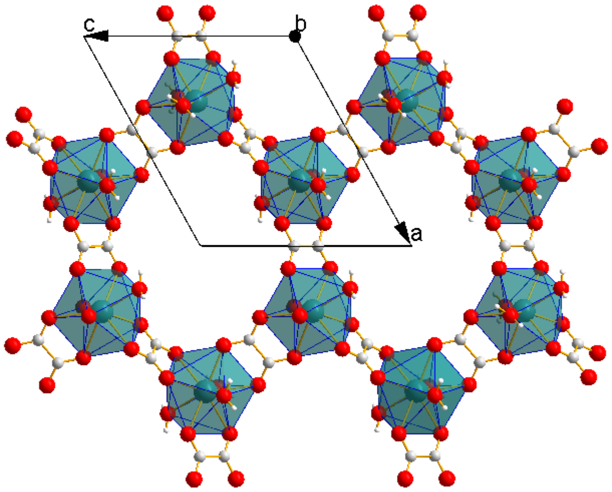

Figure S2 A hexagonal network (a "grid") in the (010) layer formed by rigid chains.

The grids in different layers are superimposed over each other with a relative shift equal to half of the lattice parameter ***c***. From Fig. 1 in the main text one can see that the chains also form layers parallel to the (100) planes. The outer-sphere water molecules are located in the voids between these layers. Based on the thermal analysis data, it can be assumed that, on dehydration, the outer-sphere water molecules are removed from the crystal structure. As a consequence, the hexagonal grid loses its stability and the chains shift along [00-1]. This crystal structure strain causes the change in the crystal shape, which we observed under optical microscopy. The length of the displacement vector and its components along and perpendicular to the ***c*** axis can be estimated from optical microscopy (see Fig. 2 in the main text). They can also be calculated using simple relations $S(//c) = a_{10}\cos(180-\beta_{10}) = 0.49a_{10} = 5.6A = 0.552c_{10}$, $S(\perp c) = a_{10}\sin(180-\beta_{10})-a_6 = 1.584Å$, where a_{10} and β_{10} are the lattice parameter ***a*** and the monoclinic angle ***β*** of the parent decahydrate crystal structure, a_6 is lattice parameter ***a*** of the hexahydrate crystal structure.

One can assume that the mutual arrangement of atoms inside the "rigid" chains do not change significantly during the transformation. On the other hand, it is obvious that the orientation of the oxalate groups that connect the "rigid" chains can and should change during the transformation. As a result of the mutual movement of the chains, these oxalate groups must turn and bend.

In order to find the atomic coordinates in the product structure, we have assumed that the crystal symmetry does not change during the transformation, i.e. space group symmetry $P2_1/c$ is preserved. Two arguments supported this assumption. First, the crystal structure deformation related to the mutual relative shifts of the rigid chains does not violate the symmetry of the crystal, preserving both *c* and 2_1 symmetry operators. Second, the study of the extinction in crossed Nicol prisms for crystals with (100) habit before and after the dehydration has showed that the extinction along the ***b*** axis of the initial crystal is preserved on dehydration. This agrees with preserving the crystal symmetry of the reaction product along the ***b*** axis.

In the parent structure of the decahydrate the centroids of all oxalate groups (the middle of the C-C bond) are located at the inversion centers. Therefore, the centers of the oxalate anions in the product structure must also be located at the inversion centers and have the same coordinates as in the original structure. This restriction facilitates the task of finding the coordinates of other atoms in the product structure. There are three symmetrically independent oxalate anions in the structure. Two of them are located in the “rigid” chain. We assumed that the arrangement of atoms around the inversion centers in the “rigid” chains does not change on dehydration. This assumption allowed us to find the coordinates of the atoms located in the chains. The deformation of the unit cell leads to a shift of the inversion centers lying in the neighboring chains. In this way it determines the mutual arrangement of the chains in the product structure. The third of the three crystallographicaly independent oxalate anions connects these rigid chains together. As we have already mentioned, the positions of the atoms in this connecting oxalate anions must change significantly as a result of the structural transformation, even though the center of the anion can be expected to remain located at an inversion center.

The algorithm to find the atomic coordinates

We used the following algorithm to find atomic coordinates:

1. Find the coordinate transformation matrix from the observed change in the crystal shape. One can determine the relative position of the crystallographic axes and the lattice parameters for the two crystal structures before and after the transformation having measured the changes in the crystal shape. The following vector equations illustrate the relationships between the basis vectors of the decahydrate (index “10”) and those of the hexahydrate (index “6”)

$a_{10} = 1.18a_6 - 0.6c_6$; $b_{10} = b_6$; $c_{10} = 1.08c_6$.

The transformation matrix (6 *J* 10) will consist of the columns formed by these vectors.

$$(6\,J\,10) = \begin{pmatrix} 1.18 & 0 & 0 \\ 0 & 1 & 0 \\ -0.6 & 0 & 1.08 \end{pmatrix}$$

2. Select two inversion centers lying in the chain: (0.5; 0.5; 0.5) and (0.5; 0.5; 0). Write out the coordinates of the atoms forming the coordination polyhedron of the samarium cation with coordinates (0.6905; 0.456; 0.3595). Oxalate anions located at the selected inversion centers connect this polyhedron with the neighbors along the chain. The coordinates are given in Table S3 and belong to the asymmetric unit of the original decahydrate structure.

3. Find the positions of the atoms of the oxalate anions lying at the inversion centers (0.5; 0.5; 0.5) and (0.5; 0.5; 0) in the new coordinate system. Shift the origin of coordinates to the

inversion center and find atomic coordinates in the coordinate system of the product structure. To do this, multiply the transformation matrix by the coordinates of the atoms of the original lattice $[6,u_6] = (6\ J\ 10)\ [10,u_{10}]$.

Move the origin of the coordinate system to the initial position and find the atomic coordinates when the coordinate system of the product is located in the initial position.

4. Find the positions of the samarium atom and of the remaining oxygen atoms in the coordination polyhedron in relation to the inversion center (0.5; 0.5; 0.5) in the new coordinate system. Take into account the deformation of the chains and the displacement of the polyhedron with respect to the inversion center (0.5; 0.5; 0.5). As a result of the transformation, the lattice parameter along the ***c*** axis decreases. “Rigid” chains are arranged along this direction and their length is reduced by 7.4%. The chain unit is equal to the distance between the two nearest polyhedra and is determined by the Sm-oxalate-Sm distance. One can assume that the length of the chain unit does not change much during the transformation and the compression of the chain is associated with a decrease in the angle between the chain units. Thus, the compression of the “rigid” chain leads to a shift of the samarium atom and its environment relative to the inversion center (0.5; 0.5; 0.5). The changes in the coordinates x and z in the new structure are associated with the deformation of the chain and can be easily found knowing the chain deformation, the length of the chain unit and the angle between the units of the chain. One can thus obtain $\Delta z = +\ 0.02$, $\Delta x = +\ 0.025$, to correct the coordinates of the samarium and oxygen atoms in the polyhedron.

5. To complete the crystal structure model of the dehydration product, it is necessary to find the position of the atoms of the oxalate group connecting the rigid chains. As the chains shift, the oxalate group rotates around the inversion center. This should lead to a rotation of the polyhedron face in the shear plane (formed by the oxalate O1, O2 atoms and the O7 atom belonging to water molecule). As a result of this rotation O2 takes the place of O1, O1 shifts to the position previously occupied by O7, and O7 occupies the position of O2. We assume that the oxalate group does not change its geometry (bond angles and bond lengths) during the transformation. The center of the C-C bond of this oxalate group is located at the inversion center (1; 0.5; 0.5). The center of the O…O segment of the oxalate anion lies at the line connecting a samarium ion and the inversion center. Knowing that the distance from the inversion center (the center of the C-C bond) to the center of the O…O segment is 1.115 Å, one can find the coordinates of the latter. The angle between the center of C-C bond, the center of the O…O segment and the O1 atom is close to 90°, and the distance between the

center of the C-C bond and the O1 atom is close to that in the oxalate anion. Taking all of these facts into account one can thus find the coordinates for C and O atoms of the third oxalate anion. In this way, we could suggest a structural model for the product of the dehydration, including the coordinates of all atoms. The proposed projection of the hexahydrate structure onto the (010) plane is shown in Figure S3; the atomic coordinates are summarized in Table S3.

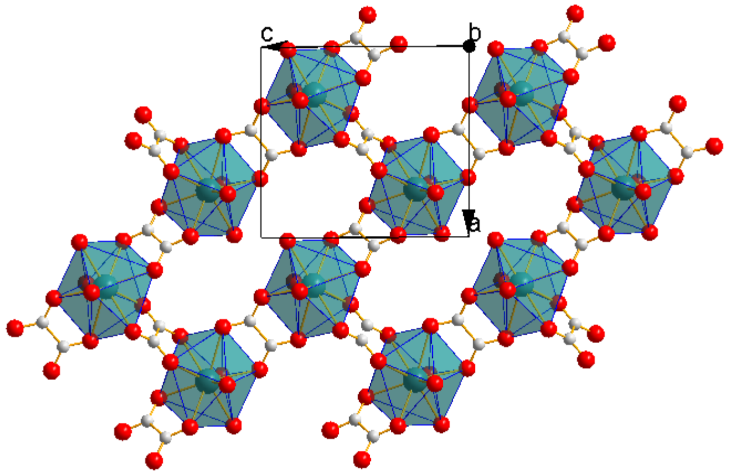

Figure S3 The proposed projection of the hexahydrate structure onto the (010) plane.

The structure of the hexahydrate can be obtained as a result of removal of all the outer-sphere water molecules and the shift of the zigzag chains along [00-1]. At the same time, the hexagonal grid becomes almost rectangular, and the monoclinic angle decreases from 118.91° to 89.5°. The geometries of the oxalate groups are almost the same in the crystal structures of the decahydrate and the hexahydrate. The distances between the samarium atom and the oxygen atoms in the polyhedron (2.3557 - 2.6025 Å) in the hexahydrate structure differ significantly as compared with those in the parent structure of the decahydrate (2.4334 - 2.5508 Å). This indicates that additional optimization of the structure is required, and can be achieved by slight turns of the oxalate groups relative to the axes that connect the center of the oxalate anion and the samarium atom. These "shuffles" allow one to optimize the crystal structure. The calculated structure is similar to that of layered lanthanide oxalate hexahydrates $[Ln(H_2O)_3]_2(C_2O_4)_3$ with Ln=Eu-Dy which have been described by (Trollet *et al.*, 1997).

We have successfully determined the coordinates of the atoms for two out of three oxalate groups, the samarium atom and O8 and O9, which belong to water molecules. This has been done by assuming that the positions of the atoms in the rigid chains are preserved. The coordinates of the third oxalate group and of water atom O7 were found based on the assumption that the polyhedron face formed by O1, O2, O7 rotates as the chains shift with respect to each other. Two types of "shuffle" can be distinguished for this structural transformation. The first type is related to the optimization of the structure and consists of a slight change in the positions of the oxalate groups of the chain, as well as

of O8 and O9 atoms of water molecules. The second type is a radical change of the position of the oxalate anion connecting the chains and the O7 atom of water molecule.

Table S3 Atomic coordinates of the asymmetric units of the initial and resulting structures*.

	$Sm_2(C_2O_4)_3{\cdot}10H_2O$			$Sm_2(C_2O_4)_3{\cdot}6H_2O$ obtained from $Sm_2(C_2O_4)_3{\cdot}10H_2O$ by dehydration		
Space group symmetry	$P2_1/c$			$P2_1/c$		
a, *b*, *c* (Å)	11.5863 (5), 9.6080 (2), 10.1371 (2)			8.55, 9.61¸ 9.39		
β (°)	118.906 (2)			89.5		
	x	y	z	x	y	z
C1	1.0006	0.5526	0.5578	0.9557	0.4449	0.5436
C2	0.5547	0.4743	-0.0161	0.5646	0.4743	-0.0502
C3	0.533	0.571	0.5305	0.5389	0.571	0.5331
O1	0.8926	0.5733	0.5547	0.8353	0.3891	0.4894
O2	0.892	0.3917	0.3553	0.989	0.58	0.3382
O3	0.6608	0.4366	0.0939	0.6897	0.4366	0.0049
O4	0.4717	0.5259	0.1523	0.4666	0.5259	0.1815
O5	0.626	0.6039	0.5077	0.6487	0.6039	0.4327
O6	0.5118	0.3574	0.401	0.5139	0.3574	0.386
O7	0.7967	0.3152	0.596	0.9826	0.3917	0.1285
O8	0.7105	0.6928	0.2838	0.7684	0.6928	0.1602
O9	0.669	0.2114	0.286	0.7194	0.2114	0.1875
Sm	0.6905	0.456	0.3595	0.749	0.456	0.2539

* - Coordinates of atoms for the parent structure were known from X-ray diffraction beforehand, coordinates of atoms for the product structure have been proposed by modelling based on the optical microscopy observations of the change in the crystal shape as a result of the dehydration.

One can see comparing Figure S3 and Figures 1 and 2 from the main text, that the crystal structure proposed based on the optical microscopy observations of the changes in the crystal shape is the same as has been determined from X-ray single-crystal diffraction.

S3. Finding the invariant plane and interface position

A martensitic transformation always preserves an invariant plane. This is a macroscopic plane that is not strained and does not change its orientation on transformation. To minimize elastic strain accompanying the transformation, the interface between the parent and the product phases must coincide with the invariant plane. The position and orientation of the invariant plane on structural transformation can be found from the analysis of pure strain. Pure strain transforms a sphere into an ellipsoid. In the directions along the principal axes of the strain ellipsoid, any line segment changes its length, but preserves orientation. Strain along the principal axes is equal to the principal components of the strain ellipsoid. Projection of the strain ellipsoid onto the (010) plane, together with the initial unit sphere, are shown in the Figure S4:

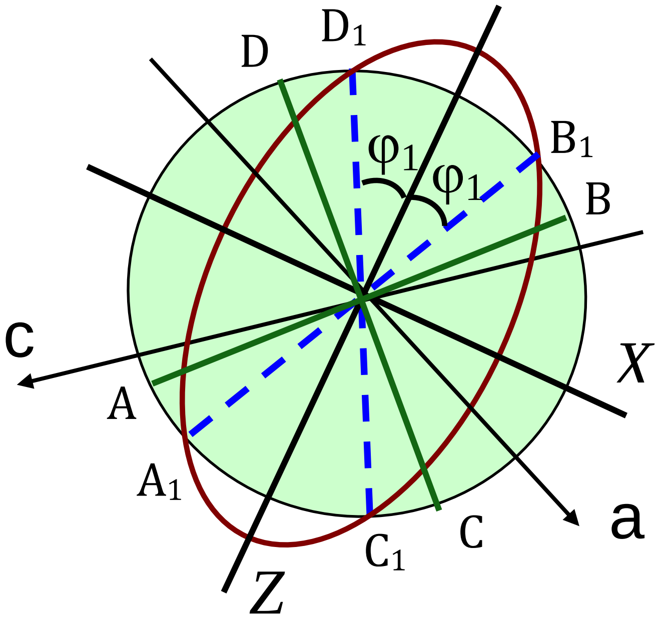

Figure S4 Projection of the strain ellipsoid onto the (010) plane, together with the initial unit sphere.

Z and X are the principal axes of the strain ellipsoid, ***a*** and ***c*** are the crystallographic axes of the parent crystal before the transformation. There are several directions in the crystal structure along which the line segments do not change their length, but change orientation over the course of the transformation. These directions coincide with the A_1B_1 and C_1D_1 lines connecting the points where the sphere and ellipsoid projections intersect each other. To find these coordinates, the following equation system must be solved:

$$\begin{cases} x_1^2 + z_1^2 = 1 \\ \dfrac{x_1^2}{(0.656)^2} + \dfrac{z_1^2}{(1.189)^2} = 1 \end{cases}$$

Solutions are the four points with coordinates (x; z): (-0.425; -0.905), (+0.425; +0.905), (-0.425; +0.905) and (+0.425;-0.905). The first two points correspond to A_1B_1, and the second two to C_1D_1. A_1B_1 and C_1D_1, are located symmetrically in relation to the X and Z axes of the strain ellipsoid. The angles between Z and these lines can be calculated as φ_1= arctan (x_1/z_1) = ±25.15°. Initial positions of these lines before deformation are for AB ((-0.648; -0.761), (+0.648; +0.761)), and CD ((-0.648; +0.761), (+0.648; -0.761)). Therefore, the angle between AB (or CD) and the Z-axis is equal to: φ = arctan (x/z) = ±40.41°. Clockwise rotation of the strain ellipsoid around Y by 15.2 ° superposes A_1B_1 with its initial position AB before deformation (Figure S5).

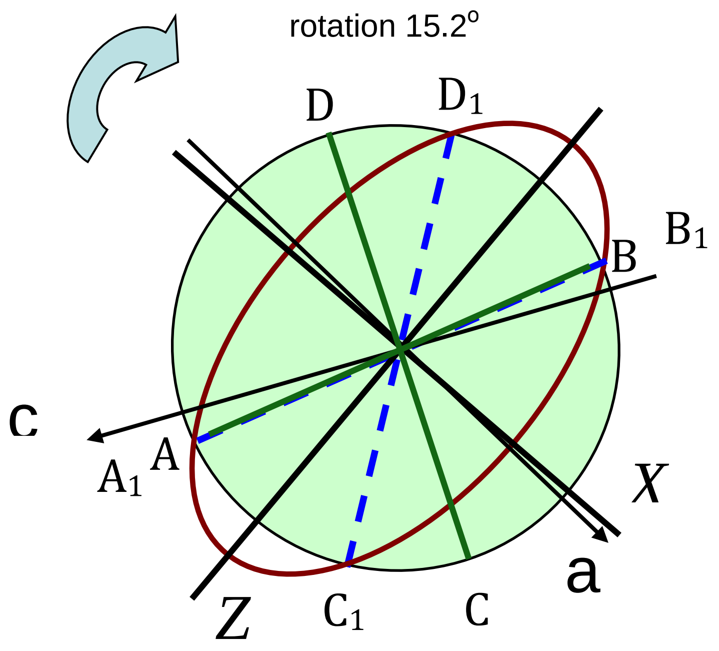

Figure S5 The invariant–line strain obtained by combining the pure deformation with a rigid body rotation.

After the pure deformation and rotation, AB does not change its length and orientation. The case corresponds to deformation with an invariant line. Since the strain along Y is small (2%), the plane formed by the AB and Y axes is an invariant plane, which does not change its orientation and is not distorted during the transformation. Pure strain combined with the rigid body rotation can generate an invariant plane, only if the two principal strain components have opposite signs and the third component's value is close to zero. For the transformation $Sm_2(C_2O_4)_3 \cdot 10H_2O \rightarrow Sm_2(C_2O_4)_3 \cdot 6H_2O$ the angle between the invariant plane and the crystallographic axis ***c*** is equal to 7.2°. The position of the interface observed experimentally matches well with the position of the invariant plane.
